# Fellow-Workers and the Roll of Honour

**Published:** 1914-11-14

**Authors:** 


### ROUND THE WORLD.

[The Editor Invites Early Information and Contributions Marked "Fellow-Workers."]

### Fellow-Workers and the Roll of Honour.

The members of the staff of Lord Tredegar's hospital-yacht *Liberty* who were not mentioned last week are the assistant surgeons, Mr. C. A. Pannett and Mr. P. Withers Green, and the assistant physician, Dr. A. Hope Gosse.

Mr. Charles Aubrey Pannett, M.D. Lond., F.R.C.S. Eng., particularly distinguished himself as a student at St. Mary's, where he was for some time surgical registrar, house surgeon, and casualty officer. No immediate opportunity of a staff appointment offering at St. Mary's, Mr. Pannett stood for election at the Royal Free Hospital, where he is now assistant surgeon. His past honours include two gold medals in London finals and first-class honours in physiology at the B.Sc. Lond.

Mr. Philip Withers Green has yet to win his laurels, having so far only had two years' experience as a qualified medical man. He gained entrance scholarships both to Epsom College and St. Mary's, and, since becoming M.R.C.S., L.R.C.P., has been house physician there, having previously held the post of demonstrator of biology in the medical school.

Dr. Alfred Hope Gosse, who, it may be noted, is anæsthetist as well as assistant physician on the *Liberty*, entered St. Mary's after doing his preliminary work at Cambridge, and qualified M.R.C.S., L.R.C.P., in 1910. He subsequently took the M.B., B.C. Cantab., and became an M.R.C.P. Lond. last year. Dr. Hope Gosse has done well at St. Mary's, where he won the Cheadle Gold Medal in clinical medicine in 1912, after which he was appointed medical registrar and junior medical tutor. He was Editor of the *St. Mary's Hospital Gazette* for awhile, and has also been house physician and resident medical officer to the inoculation department there.

Three prominent members of St. Thomas's Hospital staff are working under the British Red Cross at Netley. Of these Mr. Cuthbert Sidney Wallace is one of the assistant surgeons as well as teacher of operative surgery there. Mr. Wallace, who gained the gold medal in obstetric medicine and qualified for the gold medal in surgery at the M.B., B.S. Lond. ten years ago, has had previous experience of military surgery, and is part-author of a book describing the work of the Portland Hospital during the South African War. After qualifying he held the appointments of house surgeon, senior obstetric officer, and surgical registrar at St. Thomas's; whilst soon after becoming an F.R.C.S. Eng. he was elected to the staff of the East London Hospital for Children, which he has served for some years.

Mr. Cyril A. R. Nitch and Mr. Lionel Edward Close Norbury are at Netley with Mr. Wallace. Mr. Nitch is surgeon to out-patients at St. Thomas's and surgeon to the Evelina Hospital for Sick Children; formerly he held the appointments of demonstrator of anatomy and resident assistant surgeon at St. Thomas's, and clinical assistant to the East London Hospital for Sick Children. Mr. Nitch acquitted himself brilliantly in his professional examinations, being awarded the gold medal and University scholarship at the B.S. Lond. in 1902, the year in which he became an F.R.C.S. Eng., and later on the gold medal at the M.S. Lond. He is the author of the articles on "Gangrene," "Hare-lip," and "Cleft Palate" in Choyce's "System of Surgery."

Mr. L. E. C. Norbury, M.B. Lond., F.R.C.S. Eng., is one of the junior men at St. Thomas's, where he holds the post of demonstrator of anatomy, having lately been surgical registrar, resident assistant surgeon, and house surgeon there. He is also assistant surgeon to the Belgrave Hospital for Children, to St. Mark's Hospital, and to the Mildmay Hospital at Bethnal Green. Mr. Norbury's career as a student gave promise of future success, his academic distinctions including the Tite and Peacock Scholarship, the Cheselden Medal, the Beaney Scholarship in surgery and surgical pathology, and the Treasurer's gold medal at St. Thomas's, with honours in anatomy at the Intermediate M.B. Lond.

Mr. T. K. Dalziel, Dr. S. Gemmell, Dr. R. Muir, and Dr. R. Stockman are the lieutenant-colonels to the 3rd Scottish Hospital, General Hospital, Glasgow; whilst the following gentlemen hold the rank of major at the same establishment: Dr. J. M. Cowan, Dr. J. W. Downie, Dr. W. K. Hunter, Dr. D. Macartney, Dr. W. Maclennan, Dr. J. H. Nicoll, Dr. A. M. Ramsay, and Dr. H. Rutherfurd.

Mr. Thomas Kennedy Dalziel, M.B., C.M. Edin., F.F.P.S. Glasg., is one of the surgeons at the Western Infirmary, Glasgow, as well as a member of the staff of the Royal Hospital for Sick Children, and lecturer on clinical surgery in the University there. He has for many years enjoyed a surgical practice in Glasgow, and was at one time professor of surgery at Anderson's College and lecturer in anatomy in the Western Medical School.

Dr. Ralph Stockman, M.D. Edin., F.R.C.P. Edin., F.F.P.S. Glasg., F.R.S.E., is also a University gold medallist, and has won distinction as a physician in Glasgow, where he now holds the posts of professor of materia medica and therapeutics in the University and physician to the Western Infirmary. He has for many years been known as an indefatigable investigator of drug-actions, a subject in connection with which many interesting communications have come from his pen. At one time Dr. Stockman was one of the Research Scholars of the British Medical Association, and has examined at both Oxford and Cambridge, as well as for the I.M.S.

Dr. Robert Muir, M.D. Edin., F.R.C.P. Edin., is one of our leading pathologists and part-author of a textbook on bacteriology largely read by medical students in this country. Now professor of pathology in Glasgow University and pathologist to the Western Infirmary, Dr. Muir formerly occupied the chair of pathology at St. Andrews, previous to which he was senior demonstrator of pathology at Edinburgh and pathologist to the Royal Infirmary there. Dr. Muir has won many academic distinctions, and when qualifying was awarded first-class honours in the M.B., C.M. Edin., after which he was awarded a gold medal for his M.D. thesis.

